# A multi-source domain feature adaptation network for potato disease recognition in field environment

**DOI:** 10.3389/fpls.2024.1471085

**Published:** 2024-10-10

**Authors:** Xueze Gao, Quan Feng, Shuzhi Wang, Jianhua Zhang, Sen Yang

**Affiliations:** ^1^ School of Mechanical and Electrical Engineering, Gansu Agricultural University, Lanzhou, China; ^2^ School of Electrical Engineering, Northwest University for Nationalities, Lanzhou, China; ^3^ Agricultural Information Institute, Chinese Academy of Agricultural Sciences, Beijing, China; ^4^ National Nanfan Research Institute, Chinese Academy of Agricultural Sciences, Sanya, China

**Keywords:** field environment, potato disease recognition, multi-source unsupervised domain adaptation, multi-representation extraction, subdomain alignment

## Abstract

Accurate identification of potato diseases is crucial for reducing yield losses. To address the issue of low recognition accuracy caused by the mismatch between target domain and source domain due to insufficient samples, the effectiveness of Multi-Source Unsupervised Domain Adaptation (MUDA) method in disease identification is explored. A Multi-Source Domain Feature Adaptation Network (MDFAN) is proposed, employing a two-stage alignment strategy. This method first aligns the distribution of each source-target domain pair within multiple specific feature spaces. In this process, multi-representation extraction and subdomain alignment techniques are utilized to further improve alignment performance. Secondly, classifier outputs are aligned by leveraging decision boundaries within specific domains. Taking into account variations in lighting during image acquisition, a dataset comprising field potato disease images with five distinct disease types is created, followed by comprehensive transfer experiments. In the corresponding transfer tasks, MDFAN achieves an average classification accuracy of 92.11% with two source domains and 93.02% with three source domains, outperforming all other methods. These results not only demonstrate the effectiveness of MUDA but also highlight the robustness of MDFAN to changes in lighting conditions.

## 1 Introduction

Potato is one of the main food crops in many countries. However, various factors make them susceptible to different diseases, which can adversely affect their yield (Arshaghi et al., 2023). Early detection and warning are crucial for effective disease prevention and control, playing a pivotal role in management and decision-making (Fang and Ramasamy, 2015). In reality, it is an extremely time-consuming and unreliable way to detect and diagnose disease types by visual inspection of a farmer. Although the accuracy of laboratory-based identification method is very high, it is not suitable for the current situation of large-scale planting while the cost is high (Verma et al., 2020). This highlights the need for automated methods such as machine learning to improve the efficiency of disease recognition in agricultural environments. Given these challenges, machine learning techniques have gained increasing attention in recent years. Early machine learning methods primarily relied on manual feature extraction (Liaghat et al., 2014; Hlaing and Zaw, 2018; Deng et al., 2019; Basavaiah and Arlene Anthony, 2020). These features typically include salient structures such as texture, edges, color, and corners in the image (Sahu and Minz, 2023). Implementing such methods requires substantial engineering skills and specialized domain knowledge. Moreover, these methods are often tailored to specific problems, lacking generality.

In recent years, deep learning methods have been widely applied in crop disease recognition, achieving better performance than approaches based on manual feature extraction (Bevers et al., 2022). Atila et al. (2021) employed data augmentation techniques to augment the PlantVillage dataset to 61,486 images, evaluating the classification performance of various deep learning models. The experimental findings highlighted that the B4 and B5 models of the EfficientNet architecture achieved the highest performance in both the original and augmented datasets. Hassan and Maji (2022) introduced a novel deep learning model incorporating Inception layers and residual connectivity, achieving high classification accuracies of 99.39% on the PlantVillage dataset, 99.66% on the rice disease dataset, and 76.59% on the cassava dataset. Gu et al. (2022) proposed an enhanced deep learning-based multi-plant disease identification method, conducting experiments on 14,304 field images of six diseases in apples and pears. The results demonstrated a 14.98% improvement in accuracy compared to the baseline method.

However, deep learning, being a data-driven algorithm, heavily relies on large-scale labeled data for success. Establishing such datasets for a specific task incurs significant financial and time costs. In agricultural environments, various interfering factors pose challenges to data annotation. In addition, traditional machine learning assumes that the training data (source domain) and test data (target domain) of the model obey the independent identical distribution. However, this assumption is often invalidated in agriculture due to variables like lighting conditions, crop variety, planting environment, disease progression stages, and the tools used for data collection. The resultant disparity in distribution between the source and target domain data, known as domain shift, refers to the significant differences in data distribution encountered in transfer learning or domain adaptation (Tanabe et al., 2021). This phenomenon degrades the model’s performance, as the knowledge learned from the source domain may not generalize well to the target domain (Zhang et al., 2022).

Essentially, domain shift arises when training samples are insufficient to cover the testing ones. However, in reality, obtaining a large number of samples is prohibitively expensive, making addressing domain shift with limited samples a crucial area for exploration. Unsupervised Domain Adaptation (UDA) is one such solution. As a pivotal component of transfer learning (Pan and Yang, 2009), UDA methods aim to learn generalizable features across domains. These methods involve training a predictive function on labelled data in source domain and minimizing the prediction error on unlabelled data in target domain. UDA methods can be broadly categorized into two types (Wang et al., 2023): the first type is metric-based methods (Tzeng et al., 2014; Sun and Saenko, 2016; Zhu et al., 2020; Zhang et al., 2022), which aim to adopt a certain metric to minimize the distribution difference between the source and target domain data. The second type is adversarial-based methods (Long et al., 2015; Yu et al., 2019), which involve applying a MinMax adversarial training between a feature extractor and a discriminator to learn domain-invariant features and align the two domains.

Recently, researchers have applied UDA methods to the field of agriculture. Fuentes et al. (2021) proposed a system specifically designed for open-set learning problems, capable of performing open-set domain adaptation and cross-domain adaptation tasks. Yan et al. (2021) proposed a UDA method for cross-species plant disease recognition based on mixed subdomain alignment, building upon the Deep Subdomain Adaptation Network (DSAN), particularly addressing situations with poor correlation between the source and target domains. Extensive experiments have demonstrated that this method exhibits excellent recognition performance for subdomains with low correlation. Wu et al. (2023) used data captured in the laboratory as the source domain and field environment data as the target domain. They employed the DSAN method to align the data of each class in the two domains, achieving better classification accuracy than other UDA methods on several crop datasets.

Although these studies have made progress in applying UDA to agricultural tasks, they primarily focus on learning crop disease features from a single source domain (Single-Source Unsupervised Domain Adaptation, SUDA), overlooking the fact that real-world agricultural datasets often originate from multiple domains with diverse characteristics. This necessitates more advanced methods, such as Multi-Source Unsupervised Domain Adaptation (MUDA), to better handle such complex data. These research works, although to a certain extent, alleviates the above problems. However, they focus solely on learning the disease characteristics of crops from a single source domain (Single-Source Unsupervised Domain Adaptation, SUDA), neglecting the fact that the labelled data available in real-world scenarios originate from multiple domains. Taking potato diseases in field environment as an example, due to the constraints of capturing and labelling costs, there are very few labelled data samples conforming to the same distribution. Furthermore, the interferences of multiple factors such as capture equipment, potato varieties, planting regions, and light conditions lead to significant distribution differences among data captured under different conditions. Although images captured under different conditions contain a wealth of disease feature information, SUDA only has one source domain and cannot simultaneously utilize data from multiple sources. To prevent ‘negative transfer’, which occurs when significant differences or weak correlations between the source and target domains cause the transferred knowledge to degrade the target domain’s model performance (Wang et al., 2019), it is necessary to restrict SUDA’s selection of data, thereby exacerbating the issue of data scarcity. To effectively utilize multiple potato disease datasets characterized by significant distributional disparities, the Multi-Source Unsupervised Domain Adaptation (MUDA) method emerges as a preferred choice. The simplest way to implement MUDA is by merging all source domains into a single domain (referred to as Source Combine), followed by aligning data distributions using the SUDA approach. This approach may improve the predictive capabilities of SUDA models due to data expansion (Zhu et al., 2019a). However, it may exacerbate the mismatch problem when aligning the multiple source domains with the target domain, resulting in unsatisfactory performance of the model on the target domain (Csurka, 2017). Thus, MUDA methods have been proposed to more effectively utilize multi-source data.

Early MUDA mainly uses a shallow model combination classifier to use data from multiple source domains. Xu et al. (2018) proposed a Deep Cocktail Network (DCN) to address issues related to domain and category shifts among multiple sources. Peng et al. (2019) introduced the M3SDA method, which dynamically adjusts the moments of feature distribution to transfer knowledge learned from multiple source domains to an unlabelled target domain. The aforementioned methods can be categorized into two groups: the first involves mapping multiple source domains and the target domain into a common feature space, where their distribution differences are minimized; the second involves combining classifiers trained separately on multiple source domains to obtain the final classifier for the target domain.

**Figure 1:**
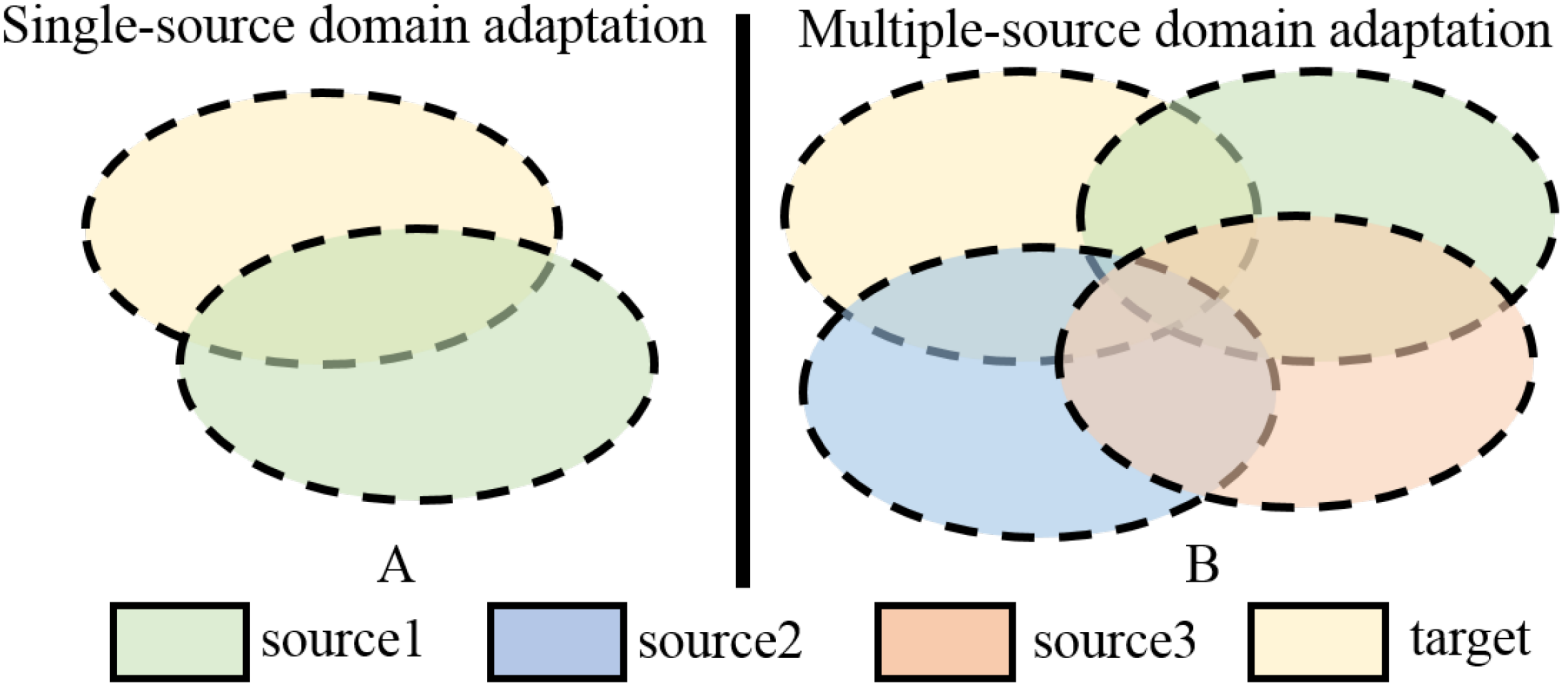
Schematic of SUDA and MUDA Methods. **(A)** Single-source domain adaptation. **(B)** Multiple-source domain adaptation.

**Figure 2:**
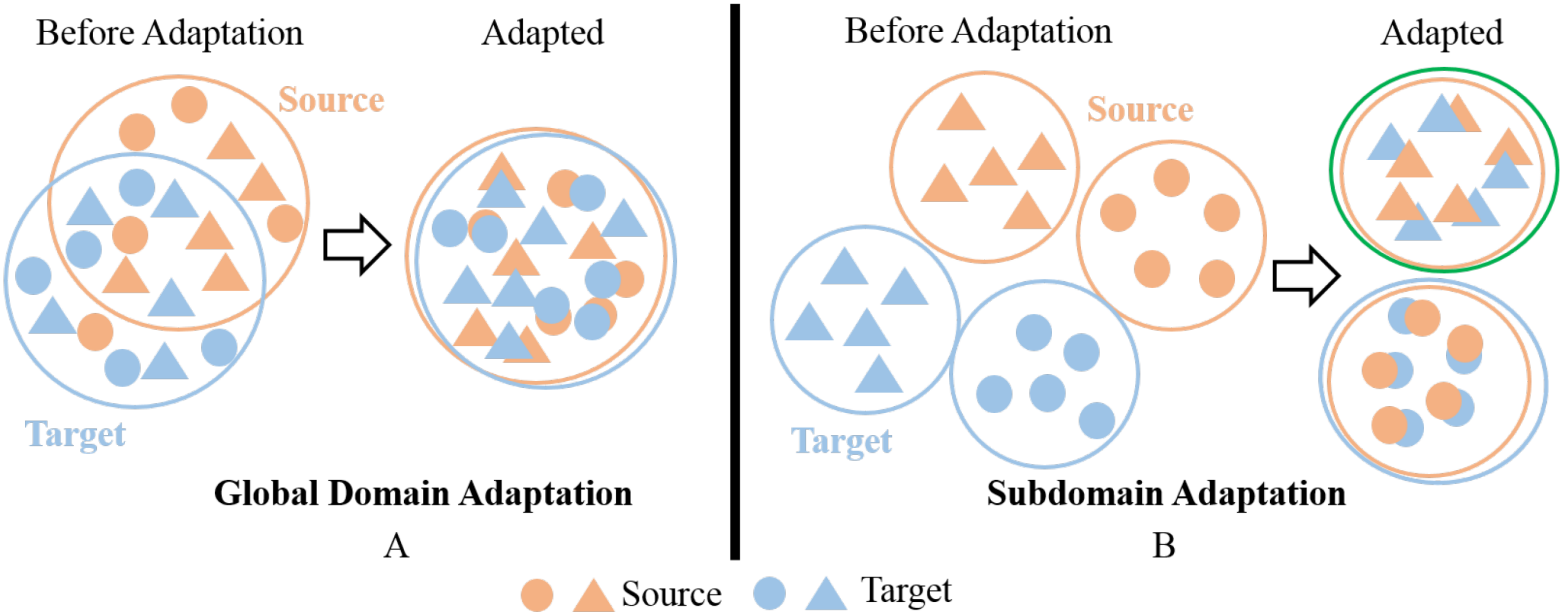
**(A)** Global Domain Adaptation **(B)** Subdomain Adaptation.

Zhu et al. (2019a) pointed out that both of these types of methods focus on extracting a common domain invariant representation for all domains, but this goal is challenging to achieve and can easily lead to significant mismatches. Even for a single source and target domain, learning their domain-invariant representations is not easy, as shown in **Figure 1A**. When attempting to align multiple source and target domains, the degree of mismatch increases and can lead to poor performance. **Figure 1B** is a schematic diagram of this process. The common domain invariant representation of the source (from source1 to source3) and target domains are their overlap. It can also be intuitively seen from the schematic diagram that when there are multiple source domains, it is very difficult to obtain their common domain-invariant representation with the target domain. Furthermore, these methods do not consider the relationship between target samples and the decision boundaries of domain-specific when matching distributions. Therefore, they proposed MFSAN to solve the above problems. While this method has shown promising results on public datasets, it still has limitations in two aspects. Firstly, when aligning each source-target domain pair, they adopted a global alignment approach, as shown in **Figure 2A**. This may lead to the failure to capture fine-grained information due to the neglect of relationships between subdomains of the same category in different domains, thereby affecting the model’s performance (Zhu et al., 2020). Secondly, the above method employed a single network structure in the feature extraction process, limiting the information that could be obtained.

While MUDA methods have demonstrated promising results in various fields such as fault diagnosis (Wen et al., 2021; Wang et al., 2022a), visual emotion classification (Lin et al., 2020), healthcare (Deng et al., 2021; Abbet et al., 2022), gait detection (Guo et al., 2021) and text sentiment analysis (Peng et al., 2023), its effectiveness in the agricultural domain has not been widely researched and validated. Wang et al. (2022b) applied the MUDA method to unsupervised crop mapping and achieved good results. Ma et al. (2023) proposed an UDA method using a multisource maximum predictor discrepancy (MMPD) neural network for county-level corn yield prediction. Case studies in the U.S. Corn Belt and Argentina demonstrate that the MMPD model effectively reduces domain differences and outperforms several other advanced deep learning and UDA methods in terms of performance. There have been no reported studies related to MUDA in the field of crop disease recognition. To address the reduction in recognition accuracy caused by complex backgrounds and significant illumination changes in potato disease recognition within field environments, we propose the Multi-Source Domain Feature Adaptation Network (MDFAN), which introduces a two-stage alignment strategy. MDFAN leverages multi-feature extraction and subdomain alignment techniques to achieve better domain alignment and feature extraction across multiple domains, resulting in more accurate potato disease recognition.

The main contributions of this paper can be summarized as follows:

1. Changes in lighting can significantly affect the RGB pixel values of images captured in field environments, causing substantial differences in image features obtained under various lighting conditions. To maximize the use of data from different lighting conditions to train disease recognition models and enhance their generalization ability and avoid negative transfer, using the MUDA method is a feasible option. This paper proposes a disease recognition model named MDFAN to achieve this goal.
2. MDFAN includes two alignment stages. The first stage is domain-specific distribution alignment, primarily aimed at achieving distribution alignment for each source-target domain pair within a specific domain. The second alignment stage is called classifier alignment. In this stage, the predictions of various classifiers are aligned through decision boundaries specific to the domain, mitigating prediction discrepancies between different classifiers and enhancing prediction consistency.
3. In the domain-specific distribution alignment stage, a multi-representation extraction module and a subdomain alignment module (as illustrated in **Figure 2B**) are employed to learn multiple representations of domain variables for source-target domain pairs. These techniques help capture finer-grained information between subdomains of the same category across different domains and achieve more effective alignment.
4. Based on the lighting conditions during image capture, the dataset is divided into four domains, each encompassing five disease types. Extensive experiments were conducted on this dataset using 2 and 3 source domains to evaluate the performance of MDFAN. The experimental results demonstrate the robust generalization capability of MDFAN under this interference.

The rest of this paper is organized as follows. Section 2 provides a detailed introduction to the experimental data and MDFAN. Section 3 validates the effectiveness of MDFAN through experiments conducted on the dataset. Finally, some conclusions are drawn in Section 4.

## 2 Materials and methods

### 2.1 Database

**Figure 3:**
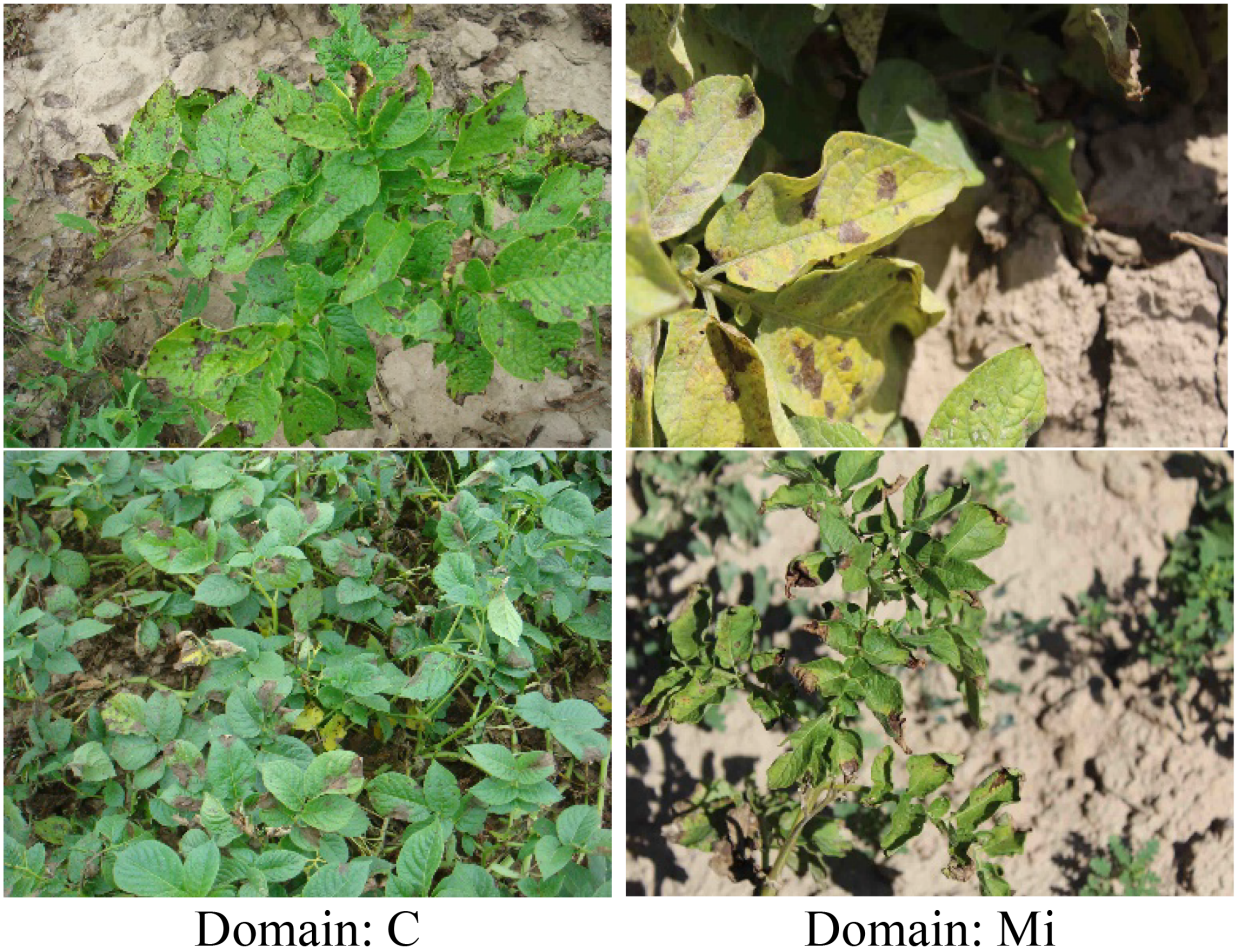
Lighting Conditions Comparison.

The study by (Suh et al., 2018) indicates that changes in illumination are a significant factor causing differences in data distribution. Therefore, to evaluate the efficacy of MDFAN under conditions where illumination is the primary variable, a potato disease dataset, named DIF_light_intensities, was constructed, with all images captured in the field. Based on the time periods of image capture and the weather conditions (sunny or cloudy), we partition the data into four domains: Morning (Mo), Midday (Mi), Afternoon (A), and Cloudy (C). Among them, the pictures in the Mo, Mi and A domains are all captured on sunny days, which makes the contrast between light and shade caused by occlusion prevalent in the pictures, and there are still some overexposed pictures in the Mi domain. In the C domain, the image is generally dark due to insufficient lighting. In **Figure 3**, we present two images each from the insufficient lighting (Domain: C) and the sufficient lighting (Domain: Mi), providing a visual comparison to highlight the lighting differences in field environments.

Each domain contains images of Potato Cercospora Leaf Spot (PCLS), Potato Early Blight (PEB), Potato Late Blight (PLB), Potato Macrophomia Blight (PMB), Potato Powdery Mildew (PPW). Each disease type corresponds to a subdomain in its own domain. Taking the Mo domain as an example, it contains five subdomains: PCLS, PEB, PLB, PMB, and PPW. Therefore, the subdomain labels in these four domains are consistent.

**Figure 4:**
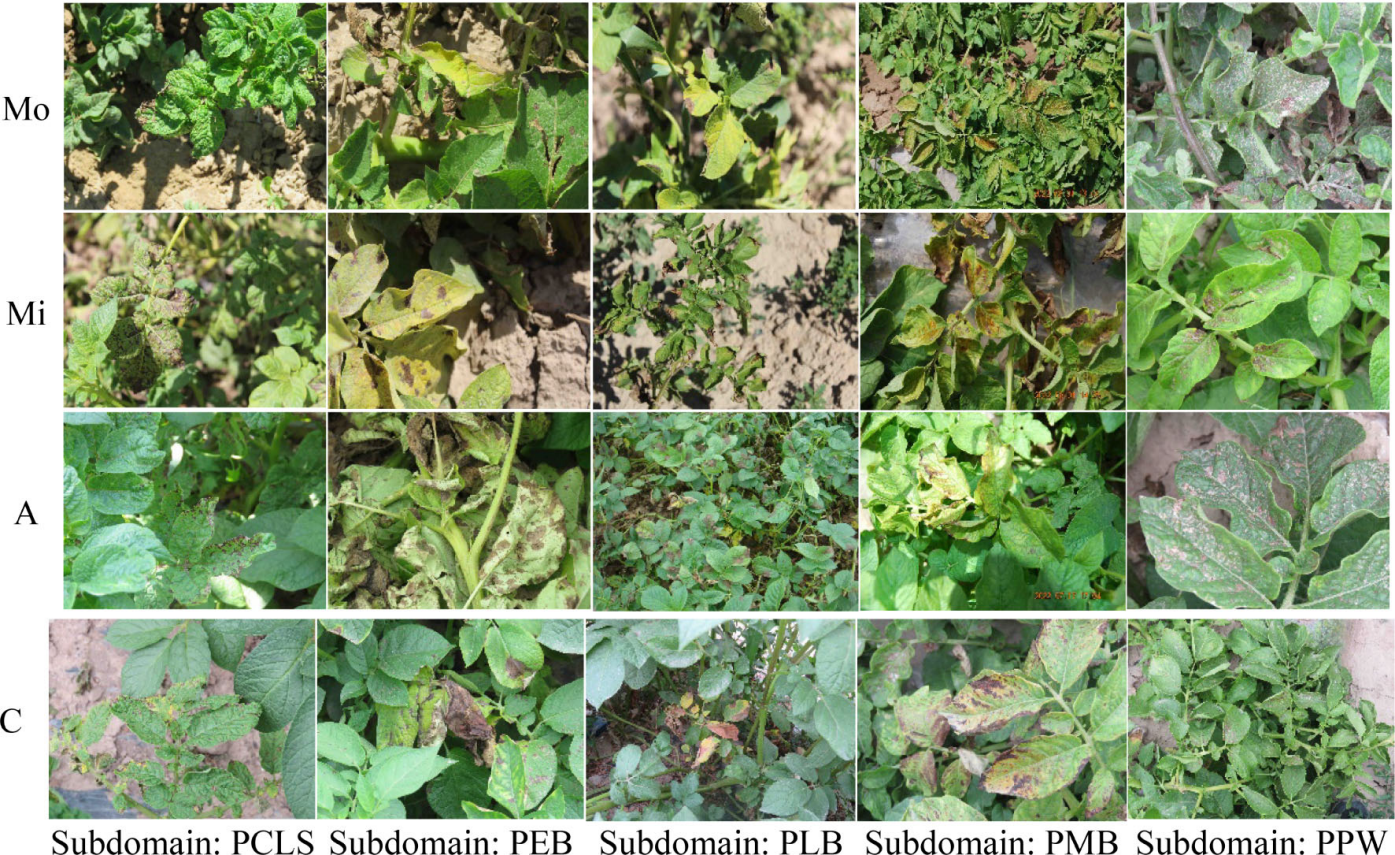
Disease Illustration in DIF_light_intensities Dataset.

The domains and corresponding subdomains of disease images in this dataset are illustrated in **Figure 4**.

**Table 1** provides relevant information about the dataset, including the capture time of images in each domain, the number of images for each disease type, and other pertinent information. Taking Mo as an example, the image capture time is 7: 00-11: 00, and there are 86 PEB images.

**Table 1:** Composition of DIF_light_intensities dataset.

Domain		Types of Diseases				
Name	Time	PCLS	PEB	PLB	PMB	PPW
Mo	7: 00 -11: 00	88	86	88	90	102
Mi	11: 30 -15: 30	87	91	96	78	96
A	16: 00 -18: 30	91	89	95	84	93
C	7: 00 -18: 30	92	74	66	46	36

To comprehensively assess the effectiveness of MDFAN, the investigation focuses on the transfer tasks associated with both the 2-source and 3-source domains of the dataset. Among them, there are twelve transfer tasks corresponding to 2-source domains, abbreviated as: Mo, Mi →A; Mo, Mi →C; Mo, A →Mi; Mo, A→C; Mi, A →Mo; Mi, A→C; Mo, C →Mi; Mo, C→A; Mi, C →Mo; Mi, C→A; A, C →Mo; A, C →Mi. Additionally, there are four transfer tasks corresponding to the 3-source domains, abbreviated as: Mo, Mi, A→C; Mo, Mi, C→A; Mo, A, C →Mi; Mi, A, C →Mo. In the case of Mo, Mi → A, where Mo, Mi represent two available source domains and A represents the target domain, the arrow “→” denotes the transfer process, and the 3-source domains follow a similar pattern.

We have devoted significant effort to obtaining potato disease data under varying lighting conditions in field environments and have developed the DIF_light_intensities dataset. This is because changes in lighting directly affect image brightness, contrast, and shadows, making it challenging for models to effectively extract features. This dataset offers a practical and challenging platform for evaluating the robustness of our method. The following section provides a detailed introduction to the MDFAN method, which is designed to mitigate the impact of lighting variations through a carefully crafted two-stage alignment strategy.

### 2.2 Methods

#### 2.2.1 Problem definition

This section introduces the proposed potato disease recognition method, MDFAN, designed for use in field environments. First, we make some basic assumptions:

1. The data in the source domain are labelled, and an effective source classifier can be constructed.
2. The target domain data is unlabelled.
3. The feature space and label space of each domain are the same, but the probability distributions differ.

In MUDA, *N* different source domains are considered, with their data distributions represented as ${\left\{\begin{array}{c} p_{sj}(x,y) \end{array}\right\}}_{j=1}^{N}$ , where *sj* denotes the *j-*th source domain. The labelled data from the source domain is denoted as ${\left\{\left(\begin{array}{c} X_{sj},Y_{sj} \end{array}\right)\right\}}_{j=1}^{N}$ , where $X_{sj}={\left\{x_{i}^{sj}\right\}}_{i=1}^{\left|X_{sj}\right|}$ refers to the samples from source domain *j*, and $Y_{sj}={\left\{y_{i}^{sj}\right\}}_{i=1}^{\left|X_{sj}\right|}$ refers to the corresponding labels. The distribution of the target domain is represented as $p_{t}(x,y)$ . Sampling from this distribution results in the target domain data, denoted as $X_{t}={\left\{x_{i}^{t}\right\}}_{i=1}^{\left|X_{t}\right|}$ , where $Y_{t}$ is unlabelled. Our aim is to establish an effective prediction model using data from multiple source domains to achieve accurate classification of target domain samples.

#### 2.2.2 Overview of network framework

**Figure 5:**
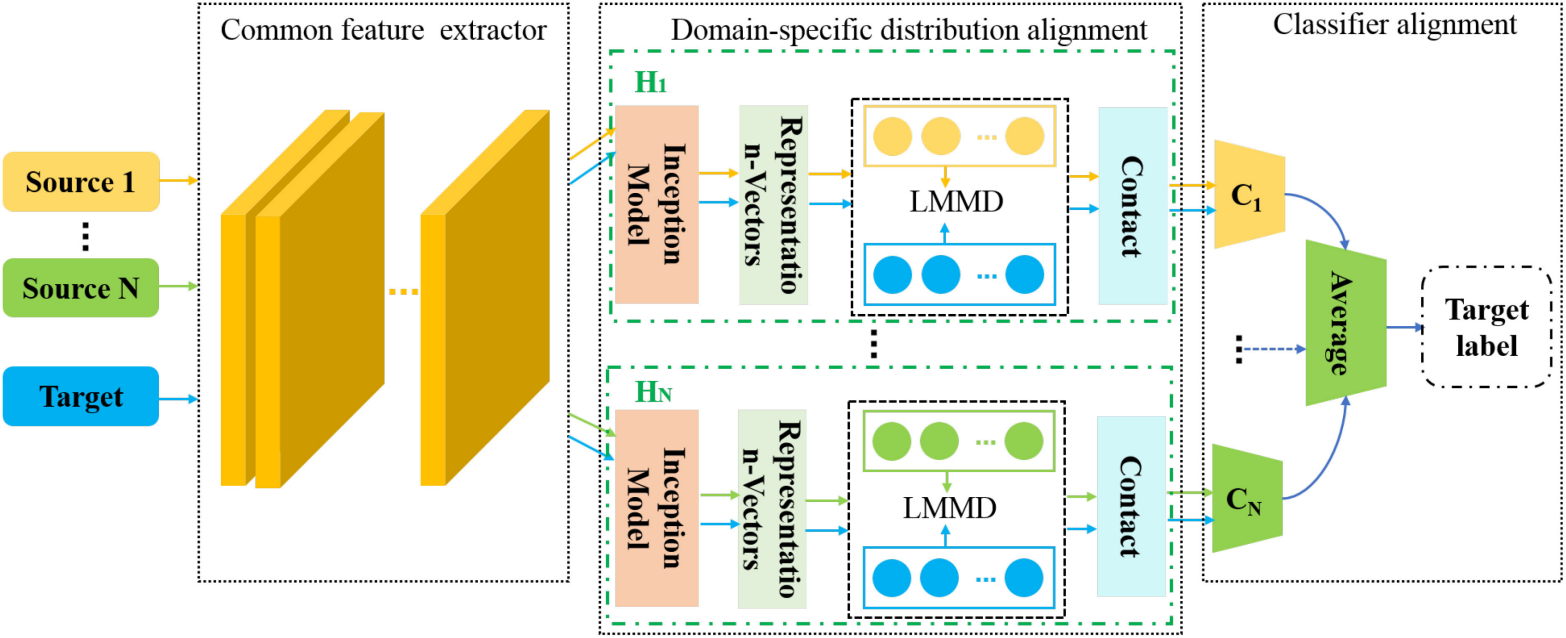
Structure of the MDFAN.

The structure of the proposed MDFAN is depicted in **Figure 5**. It consists of three parts: common feature extractor, domain-specific distribution alignment, and classifier alignment. The domain-specific distribution alignment is the first alignment stage. In this stage, the distribution alignment in the specific domain is achieved for each source-target domain pair by using multiple domain-invariant representations of the source-target domain pair. The method for extracting multiple domain-invariant representations for each source-target domain pair is to map each domain-invariant representation to a specific feature space and match their distributions. Generally speaking, the simplest way to map each source-target domain pair to a specific feature space is by training multiple networks, but this approach is extremely time-consuming. In MDFAN, this objective is achieved through the use of two subnetworks. The first part is a shared subnetwork used to learn common features across all domains, which is the common feature extractor in the MDFAN structure. The second part consists of *N* subnetworks corresponding to specific domains, also referred to as non-shared subnetworks. As shown in **Figure 5**, H*_j_* (*j*=1,…,N) represents a non-shared subnetwork, which belongs to the Source *j* -target pair, and its obtained weights are not shared. In each non-shared subnetwork, a multi-representation extraction module, referred to as the Inception Module (Szegedy et al., 2015), is used to capture more fine-grained information. In addition, in conjunction with the LMMD metric method in the subdomain alignment module, to minimize intra-class variance as much as possible. After each non-shared subnetwork, there is a corresponding domain-specific classifier *C_j_*. Due to the potential inconsistencies in the predictions of *C_j_* for target domain data near different domain decision boundaries, a classifier alignment module was designed as the second alignment stage. This module aligns the outputs of domain-specific classifiers for target samples to enhance prediction consistency.

#### 2.2.3 Common feature extractor

ResNet50 (He et al., 2016) is a classic feature extraction network architecture widely used across various tasks. In MDFAN, ResNet50 is utilized as the common feature extractor to extract shared features from all domains. Upon sequentially inputting data from Source 1 to Source *N* and the target domain sequentially, it maps images from the original feature space of each domain to a common feature space.

Let F(·) denote the ResNet50 network. Then, for a batch of images $x^{sj}$ from the source domain $\left(\begin{array}{c} X_{sj},Y_{sj} \end{array}\right)$ and a batch of images $x^{t}$ from the target domain $X^{t}$ , the common feature extraction process can be represented as follows:


(1) $$f\left(x^{sj}\right)=F\left(X_{sj}\right),f\left(x^{t}\right)=F\left(X^{t}\right)$$

Here, $f\left(x^{sj}\right)$ represents the features learned from the *j*-th source domain, and $f\left(x^{t}\right)$ represents the learned features from the target domain.

#### 2.2.4 Domain-specific distribution alignment

This part is used to implement the first alignment stage, consisting of *N* independent non-shared subnetworks $H_{j}\left(\cdot \right)$ . Each $H_{j}\left(\cdot \right)$ corresponds to a source-target domain pair, where *N* equals the number of source domains. $H_{j}\left(\cdot \right)$ contains an Inception module that learns multiple domain-invariant representations of source-target domain pairs in a specific domain. It is also called a domain-specific feature extractor. It also includes a subdomain alignment module that implements domain-specific distribution alignment. The details are introduced as follows.

(1) Domain-specific feature extraction.

**Figure 6:**
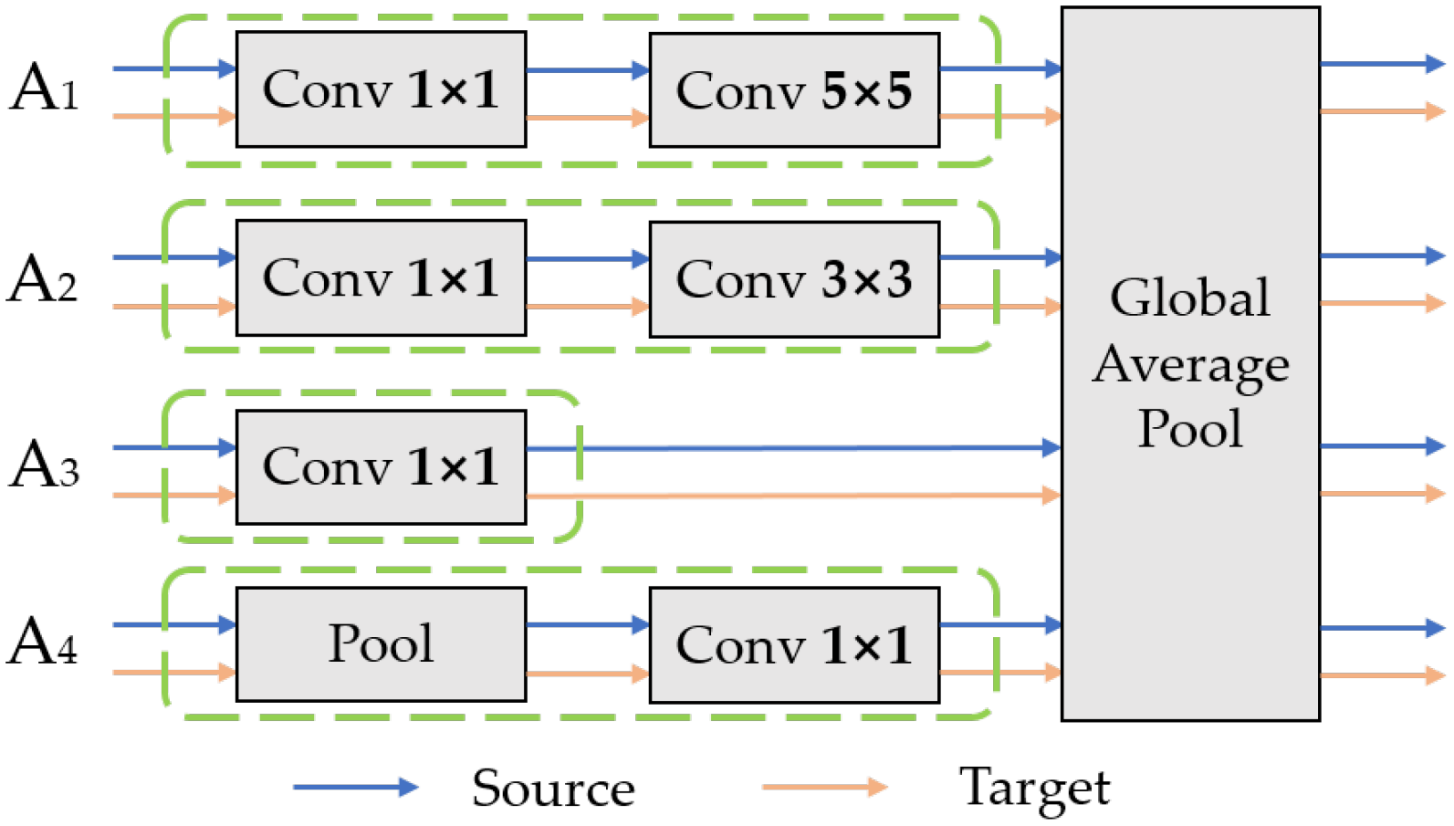
Architecture of the Inception Module.

To minimize the differences in data distribution between the source and target domains during subsequent processes, multiple domain-invariant representations of each source-target domain pair are mapped to a specific feature space. The domain-specific feature extractor is designed to achieve this goal. First, the common features $f\left(x^{sj}\right)$ and $f\left(x^{t}\right)$ are obtained from the shared feature extractor. Then, the common features of each source-target domain pair will be mapped to a specific feature space by their corresponding non-shared subnetwork $H_{j}\left(\cdot \right)$ . The complete structure of the Inception module is illustrated in **Figure 6**, consisting of a total of four branch structures: A1 - A4. This structure increases the width of the network, with each branch complementing the others, effectively capturing the overall information of the leaves in the image. In addition to acquiring overall features, it maintains the relationship between the leaves and the background environment. Taking Source1-target as an example, the features of Source1 and the target obtained from the shared feature extractor are sequentially inputted into the Inception module. The Inception module processes input data through four parallel paths, allowing different paths to independently extract features. The resulting representation vector is the specific domain feature representation corresponding to the Source1-target domain pair. The vector representations obtained from each branch are outputted to the corresponding specific domain predictor *C*_1_ after being processed by the corresponding specific domain distribution alignment module.

(2) Domain-specific distribution alignment.

To achieve better alignment in the first stage, the conventional Maximum Mean Discrepancy (MMD) (Gretton et al., 2012) is not chosen. This is because the MMD method focuses on the global distribution alignment between the source and target domains at the domain level. Its drawback is that even if the distributions of the source domain and the target domain are well aligned, errors in aligning the same subdomains in these two domains may occur. This can lead to the loss of fine-grained information, thereby affecting the model’s performance. The LMMD (Local Maximum Mean Discrepancy) method proposed by (Zhu et al., 2020) is employed, which achieves effective domain-level alignment by accurately aligning the distributions of relevant subdomains across different domains.

To achieve subdomain alignment, the source domain $D_{s}$ and the target domain $D_{t}$ are divided into *k* subdomains, $D_{s}^{\left(k\right)}$ and $D_{t}^{\left(k\right)}$ , respectively, based on the number of categories *K*. Here, *k* takes on values from the set {1,2,⋯,K} to represent category labels. In the DIF_light_intensities dataset, when the Mi domain serves as the source domain, it is split into five subdomains: PCLS, PEB, PLB, PMB, and PPW. In the case of the Mo domain as the target domain, it is also split into five subdomains.

In formal terms, LMMD defines the following divergence metric:


(2) $$d_{ℋ}(p,q)≜E_{k}{\left\|E_{p^{\left(k\right)}}[\phi (x^{s})]-E_{q^{\left(k\right)}}[\phi (x^{t})]\right\|}_{ℋ}^{2}$$

Here, $E_{k}\left[\cdot \right]$ represents the mathematical expectation of category *k*. $x^{s}$ and $x^{t}$ are instances of the source domain $D_{s}$ and the target domain $D_{t}$ , respectively. $p^{\left(k\right)}$ and $q^{\left(k\right)}$ are the distributions of $D_{s}^{\left(k\right)}$ and $D_{t}^{\left(k\right)}$ , respectively. ℋ denotes the Reproducing Kernel Hilbert Space (RKHS) equipped with the characteristic kernel κ . ϕ(·) is the mapping function that transforms the original sample into one of the RKHS feature maps. The kernel κ can be defined as $\kappa (x^{s},x^{t})=\langle \phi (x^{s}),\phi (x^{t})\rangle$ , where 〈·,·〉 denotes the inner product of vectors. By minimizing Equation 2, the gap between the distribution of the corresponding subdomains can be narrowed.

It is assumed that each sample is associated with a category according to the weight $w^{k}$ . Therefore, the unbiased estimate of Equation 2 can be expressed as:


(3) $${\hat{d}}_{ℋ}(p,q)=\frac{1}{k}\sum_{k=1}^{K}\left\|\sum_{x_{i}^{s}\in D_{s}}w_{i}^{sk}\phi (x_{i}^{s})-\sum_{x_{j}^{t}\in D_{t}}w_{i}^{\mathrm{tk}}\phi (x_{j}^{t})\right\|$$

Where $w_{i}^{sk}$ and $w_{i}^{tk}$ represent the weights of $x_{i}^{s}$ and $x_{j}^{t}$ belonging to category *k*, respectively. Both $\sum_{i=1}^{n_{s}}w_{i}^{\mathrm{sk}}$ and $\sum_{j=1}^{n_{t}}w_{j}^{tk}$ sum to 1, and $\sum_{x_{i}\in D}w_{i}^{k}\phi (x_{i})$ is the weighted sum over category *k*. The weight $w_{i}^{k}$ for the sample $x_{i}$ is calculated using the following equation:


(4) $$w_{i}^{k}=\frac{y_{ik}}{\sum_{(x_{j},y_{j})\in D}y_{jk}}\;$$

Where $y_{ik}$ is the *k*th value of the vector $y_{i}$ . For samples in the source domain, the true labels $y_{i}^{s}$ are used to calculate the weight $w_{i}^{sk}$ for each sample. However, since the target domain lacks available labels, the probability distribution predicted by the neural network is used instead. Since the output is a probability distribution, it can effectively assign $x_{i}$ to each category *K*.

After applying LMMD to the representation vectors obtained from the multi-representation module, Equation 3 can be rewritten as:


(5) $${\hat{d}}_{ℋ}(p,q)=\frac{1}{K}\sum_{k=1}^{K}{\left\|\frac{1}{n_{s}}\sum_{x_{i}^{s}\in D_{s}}w_{i}^{sk}\phi \left(x_{i}^{s}\right)-\frac{1}{n_{t}}\sum_{x_{j}^{t}\in D_{t}}w_{j}^{tk}\phi \left(x_{j}^{t}\right)\right\|}_{ℋ}^{2}$$

As ϕ(·) cannot be directly computed, it is replaced by Equation 6, resulting in the LMMD loss being redefined as:


(6) $$\begin{array}{l} {\hat{d}}_{l}(p,q)=\frac{1}{K}\sum_{k=1}^{K}[\frac{1}{n_{s}^{{\left(k\right)}^{2}}}\sum_{i=1}^{n_{s}}\sum_{j=1}^{n_{s}}w_{i}^{sk}w_{j}^{sk}\kappa \left(z_{i}^{s},z_{j}^{s}\right)\\ +\frac{1}{n_{t}^{{\left(k\right)}^{2}}}\sum_{i=1}^{n_{t}}\sum_{j=1}^{n_{t}}w_{i}^{tk}w_{j}^{tk}\kappa \left(z_{i}^{t},z_{j}^{t}\right)\\ \text{ -}\frac{2}{n_{s}^{\left(k\right)}n_{t}^{\left(k\right)}}\sum_{i=1}^{n_{s}}\sum_{j=1}^{n_{t}}w_{i}^{sk}w_{j}^{tk}\kappa \left(z_{i}^{s},z_{j}^{t}\right)] \end{array}$$

Among them, ${\left\{z_{i}^{s}\right\}}_{i=1}^{n_{s}}$ and ${\left\{z_{j}^{t}\right\}}_{j=1}^{n_{t}}$ represent the activations of the source and target subdomains samples after the multi-feature extraction, respectively, and $w^{sk}$ and $w^{tk}$ represent the weights of $z^{s}$ and $z^{t}$ belonging to class *K*.

Equation 6 can be briefly expressed as:


(7) $${ℒ}_{Lmmd}=\frac{1}{N}\sum_{j=1}^{N}\hat{d}(H_{j}(F(X_{sj})),H_{j}(F(X_{t})))$$

#### 2.2.5 Classifier alignment

During the domain-specific distribution alignment process, the results obtained from each non-shared subnetwork $H_{j}\left(\cdot \right)$ are fed into their corresponding predictor *C_j_* in the classifier alignment section. This predictor is referred to as the domain-specific predictor. Because predictors are trained independently on various source domains, they often exhibit discrepancies in their predictions for target samples. This limitation easily leads to misclassification of samples near category boundaries in the target domain. To address this issue, we propose the second alignment stage, referred to as classifier alignment. It consists of two parts: domain-specific classifiers and prediction alignment. They are introduced separately as follows.

(1) Domain-specific classifiers

The domain-specific classifier *C* is a multi-output network consisting of *N* specific domain predictors ${\left\{C_{j}\right\}}_{j=1}^{N}$ . Each predictor $C_{j}$ is a softmax classifier and receives domain-invariant features after the specific feature extractor $H_{j}(F(X_{t}))$ of the *j-*th source domain. F(·) represents the common feature extractor introduced in Section 2.2.3, while $H_{j}\left(\cdot \right)$ is the domain-specific feature extractor introduced in Section 2.2.4. Each classifier uses a cross-entropy loss, formulated as follows:


(8) $${ℒ}_{cls}=\sum_{j=1}^{N}E_{x∼X_{sj}}J(C_{j}(H_{j}(F(x_{i}^{sj}))),y_{i}^{sj})$$

(2) Prediction alignment

This stage is used to minimize the differences of the decisions among all classifiers. Specifically, by utilizing the absolute differences between the probability outputs of all predictors for target domain data as the difference loss, the calculation process is as shown in Equation 9:


(9) $${ℒ}_{disc}=\frac{2}{N\times (N-1)}\sum_{j=1}^{N-1}\sum_{i=j+1}^{N}E_{x∼X_{t}}[\left|C_{i}(H_{i}(F(x_{k})))\;-C_{j}(H_{j}(F(x_{k})))\right|]$$

In which E[·] denotes mathematical expectation, minimizing Equation 9 ensures that the probability outputs of all classifiers are similar. Finally, the average value of classifiers *C*_1_ -*C*_N_ (i.e., the ‘Average’ portion in **Figure 4**) is calculated and used as the predicted output for the target sample.

#### 2.2.6 Optimization objectives and training strategies

MDFAN includes a classifier loss ${ℒ}_{cls}$ , an LMMD loss ${ℒ}_{lmmd}$ , and a classifier discrepancy loss ${ℒ}_{disc}$ . Among them, by minimizing the classifier loss, the network can accurately classify the source domain data; minimizing the ${ℒ}_{lmmd}$ loss captures more fine-grained information, and minimizing the classifier discrepancy loss reduces differences between classifiers. Finally, the total loss is calculated as:


(10) $${ℒ}_{total}={ℒ}_{cls}+\lambda {ℒ}_{lmmd}+\gamma {ℒ}_{disc}$$

Where λ and γ are balancing parameters that compromise the roles of various functional modules. The optimization objective (10) can be easily trained and implemented using standard mini-batch stochastic gradient descent. The whole process is summarized in **Algorithm 1**.

Algorithm 1 **Multi-source domain feature adaptation network.**


1. Give the number of training epochs *T*

2. **for** *t* in 1: *T* **do**

3. Randomly pick up *m* samples 
${\left\{x_{i}^{sj},y_{i}^{sj}\right\}}_{i=1}^{m}$
 from one of *N* source domains 
${\left\{(X_{sj},Y_{sj})\right\}}_{j=1}^{N}$
.
4. Randomly pick up *m* samples 
${\left\{x_{i}^{t}\right\}}_{i=1}^{m}$
 from target domain 
$X_{t}$


5. Input the source and target samples into the common feature extractor to obtain the common latent representations 
$F(x_{i}^{sj})$
 and 
$F(x_{i}^{t})$
.
6. Input common latent representations of source samples into domain-specific feature extractor to obtain the domain-specific representations of source samples 
$H_{j}(F(x_{i}^{sj}))$
.
7. Feed domain-specific representations of the source sample 
$H_{j}(F(x_{i}^{sj}))$
 to a domain-specific classifier to obtain 
$C_{j}(H_{j}(F(x_{i}^{sj})))$
, and the classification is computed as Equation 8.
8. Feed the common latent representation of the target sample back to all domain-specific extractors, obtaining domain-specific representations for the target samples 
$H_{1}(F(x_{i}^{t1}))$
,…, 
$H_{N}(F(x_{i}^{tN}))$
.
9. Use 
$H_{j}(F(x_{i}^{sj}))$
 and 
$H_{j}(F(x_{i}^{t1}))$
 to calculate *lmmd* loss as Equation 7.
10. Use 
$H_{1}(F(x_{i}^{t1}))$
,…, 
$H_{N}(F(x_{i}^{tN}))$
to compute the *disc* loss as Equation 9.
11. Update the common feature extractor *F*, multiple domain-specific feature extractors *H_1_
*, · · ·,*H_N_
* and multiple classifiers *C_1_
*, · · ·, *C_N_
* by minimizing the total loss in Equation 10.
12. End for

## 3 Experiment

### 3.1 Baseline and comparison

At present, the commonly used method in plant disease identification is pre-training-fine-tuning. Therefore, the pre-training-fine-tuning model based on ResNet50 is used as the Baseline. Other methods participating in the comparison are summarized as follows:

(1) DDC, DAN, and DeepCoral: These three methods are commonly used metric-based approaches and are widely employed in SUDA methods. DDC (Tzeng et al., 2014) reduces domain shift between the source and target domains through the MMD, while DAN (Long et al., 2015) achieves this by using an enhanced version of MMD called MK-MMD. DeepCoral (Sun and Saenko, 2016) aligns the second-order statistics of the distributions between the source and target domains through a linear transformation.

(2) DANN, DAAN: Both of these methods are based on adversarial UDA approaches. DANN (Ganin et al., 2016) employs an adversarial learning strategy to achieve cross-domain feature fusion, while DAAN (Yu et al., 2019) shares a similar base network with DANN. The core of DAAN lies in the introduction of a conditional domain discriminator block and integrated dynamic adjustment factors, enabling dynamic adjustment of the relationship between marginal and conditional distributions.

(3) MRAN: The method (Zhu et al., 2019b) introduced a domain adaptation algorithm based on multiple representations, utilizing a hybrid structure for the extraction and alignment of multiple representations.

(4) MFSAN: Zhu et al. (2019a) integrated the technical approaches of two previous MUDA methods, achieving both the minimization of feature space differences between multiple source and target domains, and the optimization of multiple classifier outputs.

Since most of the previously mentioned methods are specifically designed for SUDA, for convenient comparison, we devise three MUDA evaluation criteria tailored for different purposes. Each criterion is introduced separately below:

(1) Single-Best: This criterion assesses the performance of a single source domain in transferring to the target domain and reports the highest accuracy achieved in the transfer task. For example, in the transfer tasks Mo, Mi → A, this criterion lists the highest accuracy results from the two transfer tasks Mo → A and Mi → A.

(2) Source-Combine: In this criterion, multiple source domains are merged, and the accuracy of each method is reported on the corresponding transfer tasks for the merged dataset. This approach can be viewed as a form of data augmentation for a single-source domain. Similarly, taking Mo, Mi →A as an example, under this criterion, it refers to merging the source domains Mo and Mi into a single source domain based on the same categories, and then listing the classification accuracy of their transfer to the target domain D.

(3) Multi-Source: This criterion is used to report the results of the MUDA method in each task. Still taking Mo, Mi →A as an example, under this criterion, the Mo and Mi domains are keep independent as source domains, and the classification accuracy of their transfer to the target domain A is listed.

The first criterion functions as a benchmark to assess whether the introduction of data from additional source domains can lead to improved accuracy in various transfer tasks, irrespective of whether it involves source domain merging or multiple source domains. The second criterion aims to demonstrate the research value of MUDA methods by evaluating their effectiveness through the merging of multiple source domains and assessing performance on corresponding transfer tasks. The third criterion is to demonstrate the effectiveness of MFSAN and MDFAN.

### 3.2 Implementation details

All experiments in this paper are conducted on the server with the following configuration: Ubuntu 18.04 system, i7-10000 processor, NVIDIA GeForce GTX 3070Ti, 8GB RAM, and PyTorch1.7 as the deep learning framework. During data loading, images are initially resized to 256x256 and subsequently randomly cropped to 224x224 before being fed into the network.

The initial learning rate is set to 0.01, batch size to 32, and the training runs for 100 epochs. Stochastic Gradient Descent (SGD) with a momentum of 0.9 is employed as the optimizer. The learning rate is adjusted during SGD using a decay strategy with the following formula: $\eta_{k}=\eta_{0}/{\left(1+\alpha \rho \right)}^{\beta }$ , where ρ linearly changes from 0 to 1 over the course of training. The initial value $\eta_{0}$ is set to 0.01, α and β are set to 10 and 0.75, respectively. In the early stages of the training process, to suppress noisy activations, λ and γ are set as dynamic adjustment factors, gradually transitioning from 0 to 1 through the following formula: $\gamma_{p}=\lambda_{p}=2/exp(-\theta p)-1$ is fixed at 10 throughout the entire experiment.

### 3.3 Experimental results and analysis

#### 3.3.1 Performance comparison

As described in Section 2.1, significant differences exist in the distribution of images captured under varying illumination intensities. Considering this variability, the DIF_light_intensities dataset is created, which accounts for changes in illumination during image capture. This dataset encompasses four domains: Mo, Mid, A, and C, each representing five distinct types of potato diseases. To evaluate the resilience of the MDFAN method against lighting variations, a comprehensive series of transfer experiments is conducted using this dataset across 2-source and 3-source domains. Each experiment is assessed using three indicators: Single-Best, Source-Combine, and Multi-Source. Detailed descriptions of these experiments are provided in the following section.

(1) 2-source domain

**Table 2** presents the classification accuracy of various algorithms on individual transfer tasks and the average accuracy across all tasks, denoted as Avg1, when using 2-source domains. The highest accuracy for each transfer task is highlighted in bold, and the second is underlined. Analyzing these values reveals several patterns. For the same SUDA method, the accuracy in the Source-Combine scenario surpasses that in the Single-Best scenario. Additionally, the Multi-Source scenario outperforms Source-Combine. These improvements can be attributed to different factors. The enhancement in Source-Combine performance results from the increased data volume after merging the source domains. In contrast, Multi-Source not only benefits from the increased available data but also effectively mitigates domain shift between different source domains and between the source and target domains.

**Table 2:** Classification accuracies (%) with 2-source domain.

Standard	Method	Mo,Mi→A	Mo,Mi→C	Mo,A→Mi	Mo,A→C	Mo,C→Mi	Mo,C→A	Mi,A→Mo	Mi,A→C	Mi,C→Mo	Mi,C→A	A,C→Mi	A,C→Mo	Avg1
Single-Best	Baseline	70.09	62.36	75.55	66.97	75.55	70.09	79.01	66.97	79.01	69.86	70.04	76.07	71.80
	DDC	56.07	51.73	67.40	54.21	67.4	56.07	61.40	54.21	61.40	54.56	58.15	60.02	58.55
	DAN	81.75	75.51	87.00	75.51	76.65	81.75	85.70	70.67	85.14	71.96	87.00	85.70	80.36
	DeepCoral	76.16	71.13	84.36	72.28	84.36	76.16	83.52	72.28	82.39	73.13	80.39	83.52	78.31
	DANN	87.38	76.67	92.07	76.91	87.88	80.84	90.74	76.91	88.71	87.38	92.07	90.74	85.69
	DAAN	79.20	72.97	78.63	76.44	79.73	79.20	84.42	76.44	85.77	77.81	79.73	85.77	79.68
	MRAN	80.61	75.29	90.53	75.29	89.97	82.24	89.61	73.90	89.61	82.24	90.53	86.91	83.89
	Avg2	75.89	69.38	82.22	71.09	80.22	75.19	82.06	70.20	81.72	73.85	79.70	81.25	–
Source-combine	Baseline	76.17	69.52	79.07	67.21	73.57	81.78	86.00	73.44	86.46	81.07	74.23	84.42	77.75
	DDC	67.99	65.43	68.72	58.89	64.54	69.16	71.11	60.97	70.43	65.65	67.62	67.72	66.52
	DAN	89.25	81.98	93.83	80.60	89.86	85.04	91.87	79.67	93.22	89.48	92.29	91.42	88.21
	DeepCoral	81.76	75.06	87.00	78.06	85.90	80.61	90.51	76.90	92.10	85.75	85.24	89.84	84.06
	DANN	92.52	78.98	94.93	77.59	92.29	89.95	92.09	72.89	94.13	90.88	94.93	92.35	88.63
	DAAN	82.24	72.75	88.11	77.82	86.13	82.01	86.90	78.06	91.87	85.74	83.26	90.06	83.75
	MRAN	83.41	79.21	89.21	77.60	88.77	83.41	89.62	76.91	89.62	87.62	88.55	88.49	85.20
	Avg2	81.91	74.70	85.28	73.97	83.01	81.71	86.41	74.12	88.04	83.74	83.73	85.97	–
Multi-Source	MFSAN	92.76	84.30	**97.14**	84.06	94.27	88.32	93.68	80.83	**94.36**	93.93	95.81	92.78	91.02
	MDFAN	**94.39**	**87.07**	96.26	**84.30**	**95.37**	**90.42**	**95.03**	**84.06**	94.13	**94.63**	**96.70**	**93.00**	92.11
	Avg2	93.58	85.69	96.70	84.18	94.82	89.37	94.36	82.45	94.25	94.28	96.26	92.89	–

Bold indicates the highest accuracy, underlining marks the second-highest. Red highlights the lowest three Avg2 values, and blue highlights the highest three.

All UDA methods, except for DDC, outperform Baseline in both individual task accuracy and average accuracy Avg1. Source-Combine demonstrates a notable improvement of 1.31%-7.97% in average accuracy compared to Single-Best. Multi-Source exhibits an even more substantial increase of 2.39%-8.36% over Source-Combine and achieves a remarkable 5.33%-13.8% improvement over Single-Best in terms of average accuracy. Based on these results, the following conclusions can be drawn: (1) Since images captured under different illumination intensities exhibit significant differences, commonly used pre-training-fine-tuning method have poor generalization ability on such experimental configuration. This results in the Baseline model having low classification accuracy. (2) The UDA method can effectively improve the accuracy of disease classification on such condition. (3) Compared to the “rudely” approach of source domain combination, Multi-Source further enhances network performance by more “sophisticatedly” leveraging data from multiple source domains.

DDC exhibits the lowest performance, and analysis suggests that this is primarily due to the characteristics of DDC’s own network structure. DDC fixes the first 7 layers of AlexNet and incorporates MMD as an adaptive metric on the 8th layer. However, AlexNet tends to overfit on small datasets, and its limited depth hinders effective extraction of abstract features.

Across twelve transfer tasks, MDFAN achieves the highest accuracy in ten of them. For individual task accuracy, it outperforms MFSAN by 0.22%-3.23%, with an average accuracy improvement of 1.09%. This demonstrates that MDFAN addresses the issue of domain shift caused by illumination changes more effectively than MFSAN. The improvement in accuracy indicates that MDFAN’s two-stage alignment strategy offers greater deployment advantages in field environments with significant lighting variations.

To analyze how the Mo, Mi, A, and C domains, and their corresponding illumination conditions, affect model performance, Avg2 is introduced, representing the average accuracy of various methods in the same transfer task. As shown in **Table 2**, Avg2 is calculated under three criteria: Single-Best, Source-Combine, and Multi-Source. The three transfer tasks with the bottom Avg2 values are marked in red, while the three with the top values are marked in blue. Under the three criteria, the bottom 3 transfer tasks of Avg2 consistently have C domain as the target domain regardless of changes in their source domains. For the top 3 tasks of Avg2, under Single-Best criterion, the target domain is consistently Mo; under Source-Combine criterion, two have Mo as the target domain and one has Mi. Under Multi-Source criterion, two have Mi as the target domain and one has Mo. Analyzing Avg2 across different modes reveals that for Source-Combine, even the worst performance is improved by 4.59% compared to Single-Best, and the best performance is enhanced by 5.82%. In contrast, Multi-Source demonstrates improvements of 13.07% and 14.48% for the worst and best Avg2 values respectively, compared to Single-Best, and 8.48% and 8.66% compared to Source-Combine. It is MDFAN that achieves this level of accuracy, further highlighting its strong robustness to illumination changes.

Based on the above results, the following conclusions can be drawn: (1) Insufficient lighting conditions, such as overcast days, can limit the model’s ability to extract features from images captured under such conditions. (2) Images captured during the morning and noon on sunny days are beneficial for improving the model’s performance. (3) Compared to single-source domain and source combine, MUDA methods demonstrate stronger robustness to varying lighting conditions.

The confusion matrix is a commonly used tool to assess classification task performance. It allows for further evaluation of the recognition accuracy of a classification model for different diseases. Each disease type corresponds to a subdomain within its respective domain. Thus, by examining the classification accuracy of each method for each disease type in the confusion matrix of a given transfer task, the strengths and weaknesses of each method can be clearly identified.

**Figure 7:**
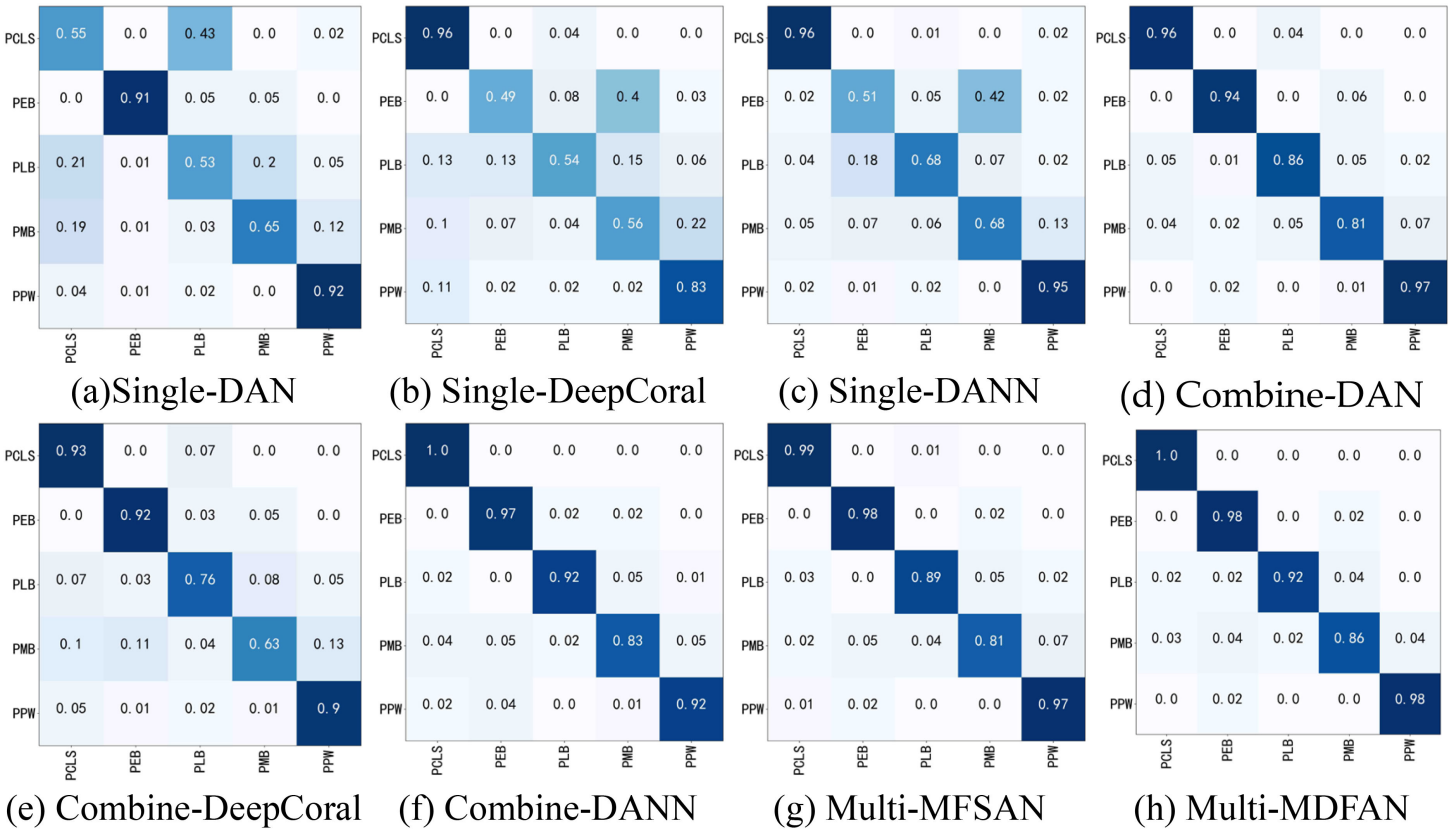
Confusion matrices of transfer task Mo, Mi → A. **(A)** Single-DAN, **(B)** Single-DeepCoral, **(C)** Single-DANN, **(D)** Combine-DAN, **(E)** Combine-DeepCoral, **(F)** Combine-DANN, **(G)** Multi-MFSAN, **(H)** Multi-MDFAN.

**Figure 7** illustrates the confusion matrices for DAN, DeepCoral, DANN and MFSAN under three criteria for the Mo, Mi → A tasks. From this figure, it can be observed that the different UDA algorithms exhibit distinct error sources when identifying potato diseases. ‘Single-’, ‘Combine-’, and ‘Multi-’ in **Figure 7** indicate that the method belongs to ‘Single-Best’, ‘Source-Combine’, and ‘Multi-Source’, respectively. The same is true in other figures. For example, the recognition rate of Single-DAN for PCLS is very low only 55% and the test samples are misidentified as PLB. The other methods in Source-Combine and Multi-Source greatly improve the accuracy of the disease. The accuracy of Combine-DANN and Multi-MDFAN is the highest, reaching 100%. The classification of the other four disease types by each method also conforms to this trend, so it is no longer repeated.

(2) 3-source domain

The results for the 3-source domains are presented in **Table 3**, from which conclusions similar to those for the 2-source domains scenario can be drawn. However, there are some differences between the experimental results of the 3-source domains and the 2-source domains, which are described below: (1) Due to the further increase in available data, the classification accuracy on individual tasks and average accuracy of each method in Single-Best, Source-Combine, and Multi-Source have improved compared to the 2-source domains scenario. (2) Under the Source-Combine criterion, the adversarial-based DANN method achieves the highest accuracy among all methods in two transfer tasks. This implies that an increase in the dataset size to a certain extent (in this experiment, the number of images for each disease type ranges from 200 to 300) is beneficial for the performance improvement of the DANN method.

**Table 3:** Classification accuracies (%) with 3-source domain.

Standard	Method	Mo,Mi,A →C	Mo,Mi,C →A	Mo,A,C →Mi	Mi,A,C →Mo	Avg
Single-Best	Baseline	66.97	70.09	75.55	79.01	72.91
	DDC	54.21	56.07	67.40	61.40	59.77
	DAN	75.51	81.75	87.00	85.70	82.49
	DeepCoral	72.28	76.16	84.36	83.52	79.08
	DANN	76.91	87.38	87.88	90.74	85.73
	DAAN	76.44	79.20	79.73	85.77	80.29
	MRAN	75.29	82.24	90.53	89.61	84.42
	Avg2	71.09	76.13	81.78	82.25	
Source-combine	Baseline	73.44	82.94	80.84	87.13	81.09
	DDC	61.43	74.30	69.84	75.62	70.30
	DAN	81.75	92.99	95.15	92.55	90.61
	DeepCoral	79.21	89.49	90.75	91.65	87.78
	DANN	79.21	**94.39**	95.37	**94.58**	90.89
	DAAN	79.91	88.08	90.75	91.87	87.65
	MRAN	75.29	85.51	86.78	89.16	84.19
	Avg2	75.75	86.81	87.07	88.94	
Multi-Source	MFSAN	85.22	93.93	94.71	93.68	91.89
	MDFAN	**87.53**	94.16	**95.81**	**94.58**	93.02
	Avg2	86.38	94.05	95.26	94.13	

Bold indicates the highest accuracy for each transfer task, underlining represents the second-highest accuracy. Red font indicates the lowest Avg2 value, and blue font indicates the highest Avg2 value.

When 3-source domain datasets are available, a similar analysis is conducted to assess the impact of the Mo, Mi, A, and C domains on the model’s performance, following the same analysis as with the 2-source domain scenario. The difference is that only the lowest value in Avg2 is marked in red, and only the best is marked in blue. From **Table 3**, it can be observed that when the target domain is C, the Avg2 is the lowest, while it is the highest when the target domain is Mi. This leads to the conclusion that insufficient lighting can affect model performance, while abundant lighting contributes to the improvement of model performance.

Although insufficient illumination adversely affects the model’s performance, the Multi-Source method can better solve this problem and improve the accuracy of the corresponding tasks. In this scenario, MFSAN achieves an accuracy of 85.22% in the task Mo, Mi, A→C, while MDFAN further improves the accuracy to 87.53%. Compared to the three transfer tasks Mo, Mi → C, Mo, A → C, and Mi, A → C with C as the target domain under the condition of 2 source domains, MDFAN achieves higher accuracies of 0.46%, 3.23%, and 3.47% respectively for the transfer task Mo, Mi, A → C. In summary, it is evident that by incorporating a third source domain, MDFAN further enhances accuracy. This demonstrates its ability to more effectively leverage diverse data sources, thereby improving the model’s generalization capability.

**Figure 8:**
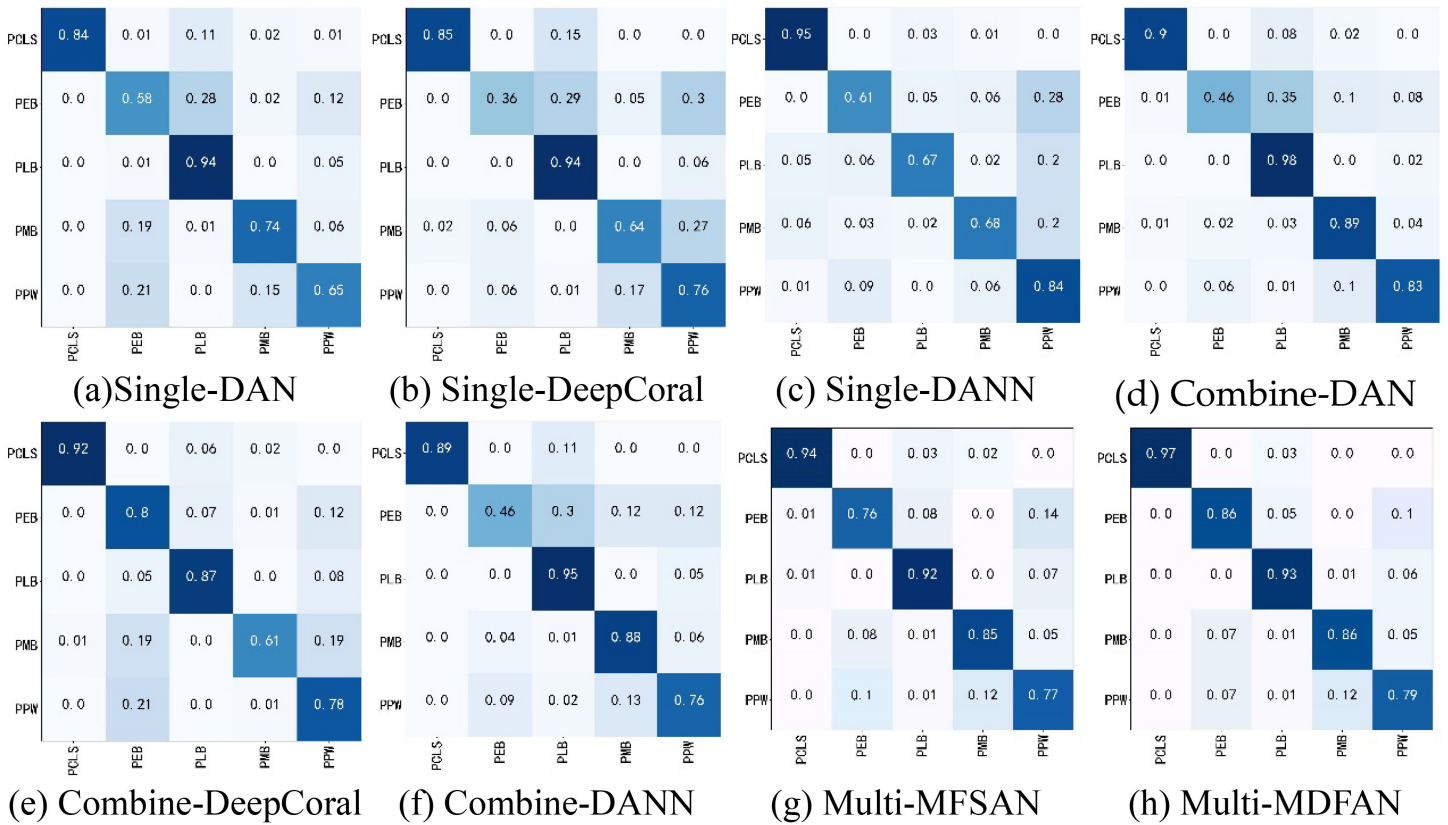
Confusion matrices of transfer task Mo, Mi, A → C. **(A)** Single-DAN, **(B)** Single-DeepCoral, **(C)** Single-DANN, **(D)** Combine-DAN, **(E)** Combine-DeepCoral, **(F)** Combine-DANN, **(G)** Multi-MFSAN, **(H)** Multi-MDFAN.

To analyze the classification of each disease by typical methods under three criteria when the target domain is C, we present their confusion matrices in **Figure 8** for the transfer task Mo, Mi, A→C.

For example, the recognition rates of Single-DAN for PEB, PMB, and PPW are 58%, 74%, and 65%, respectively. In comparison, the classification results of various methods in Combine-Source for these items show both improvements and declines in accuracy. However, the methods in Multi-Source have greatly improved the accuracies for these three diseases. The situation for the other two disease types is similar and will no longer be repeated. Overall, Multi-Source is better adapted to insufficient lighting conditions compared to Combine-Source.

#### 3.3.2 Feature visualization

To visualize the differences in the performance of UDA methods, t-distributed stochastic neighbor embedding (t-SNE) and gradient-weighted class activation mapping (Grad-CAM) are used to display the relevant results. Firstly, t-SNE is used to visualize the latent feature space representation of the source domains and the target domain, and their categories for the transfer task Mi, A →Mo in the methods DAN and DANN from Single-Best and Source-Combine, as well as MFSAN and MDFAN from Multi-Source. Since Single-Best and Source-Combine both belong to SUDA methods, and the final result of Multi-Source is obtained based on classifiers for multiple source domain-target domain pairs, the Multi-Source are further divided into single source domains for visualization. In other words, within the MUDA method, Mi, Mo → A is separately visualized by breaking it down into Mi → A and Mo → A.

**Figure 9:**
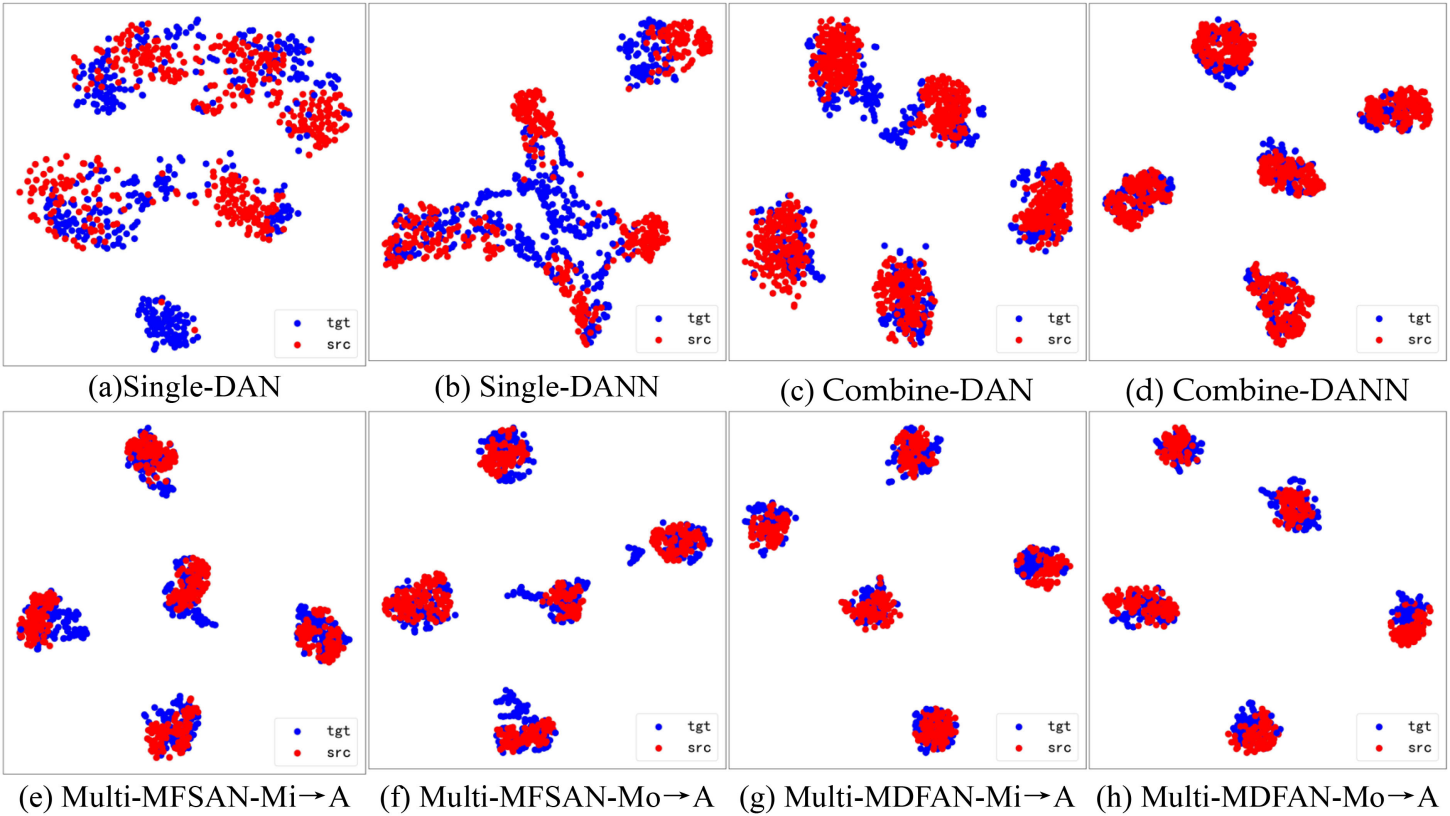
Visualization of potential spatial representations for Mi, A → Mo task using t-SNE(• is source domain, • is target domain). **(A)** Single-DAN, **(B)** Single-DANN, **(C)** Combine-DAN, **(D)** Combine-DANN, **(E)** Multi-MFSAN-Mi→A, **(F)** Multi-MFSAN-Mo→A, **(G)** Multi-MDFAN-Mi→A, **(H)** Multi-MDFAN-Mo→A.

**Figure 10:**
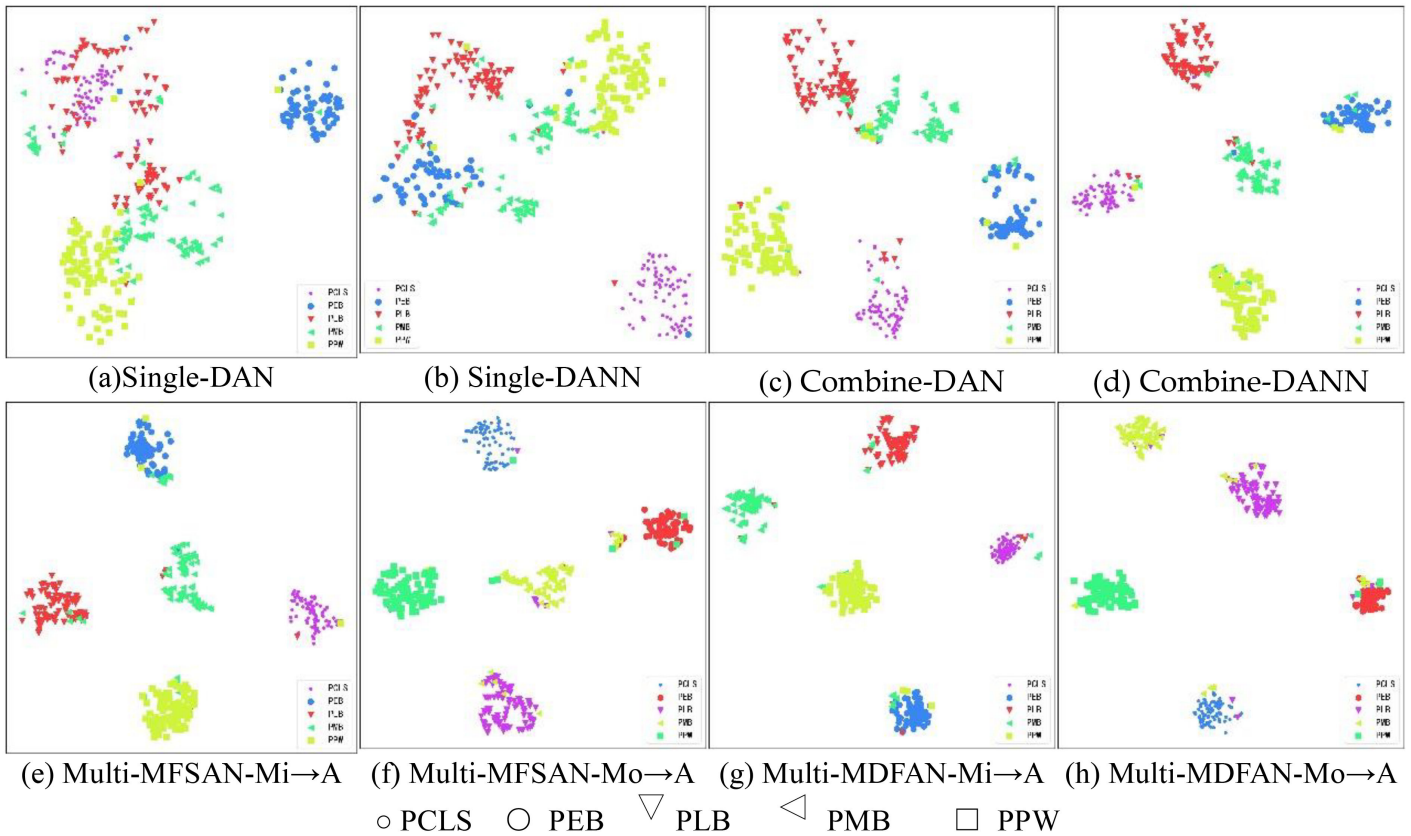
Visualization for Mi, Mo → A task using t-SNE. **(A)** Single-DAN, **(B)** Single-DANN, **(C)** Combine-DAN, **(D)** Combine-DANN, **(E)** Multi-MFSAN-Mi→A, **(F)** Multi-MFSAN-Mo→A, **(G)** Multi-MDFAN-Mi→A, **(H)** Multi-MDFAN-Mo→A.

**Figure 9** displays the feature alignment performance of the mentioned methods on this transfer task. It is evident that MDFAN has the best performance, followed by MFSAN, and DAN performs the worst. This aligns with the quantitative analysis results in **Table 2**. **Figure 10** shows the t-SNE visualization effect of the above method on the classification objects in the target domain. Different disease types are represented by various shapes and colors. It is easy to see that MDFAN has better category discrimination ability than MFSAN, DANN, and DAN. This indicates that the comprehensive use of two-stage alignment strategy, multi-representation extraction module and subdomain alignment method can make the model have better generalization ability for datasets with domain shift caused by light variation.

**Figure 11:**
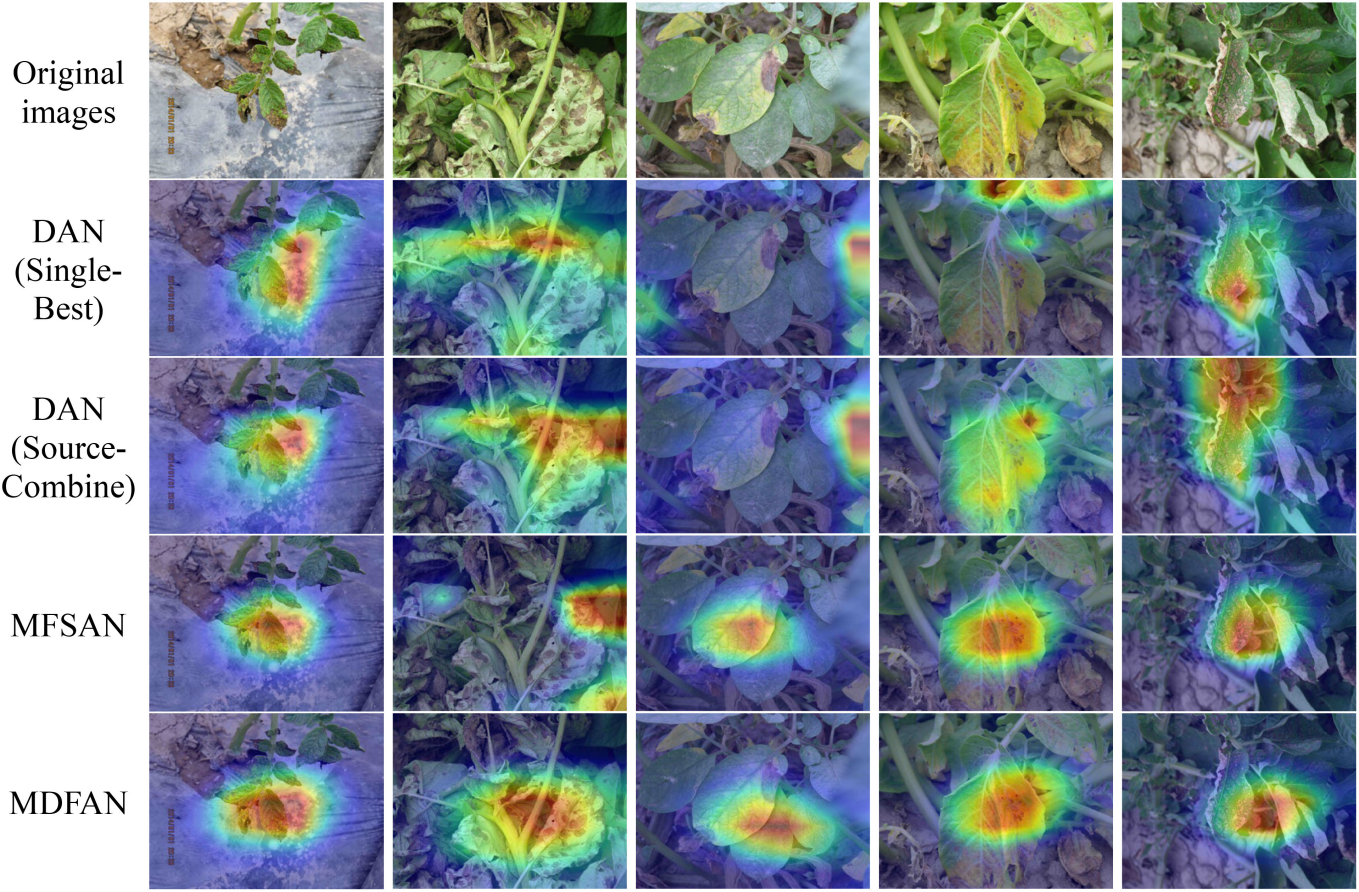
Grad-CAM visualization for Mi, Mo →A task.

In **Figure 11**, the Grad-CAM maps of several methods on the target domain A are presented. From these Grad-CAM maps, the regions of interest for each method in the images of each disease type are clearly visible. The performance of each method can be assessed based on the degree of agreement between the regions of interest and the lesion regions. The first row shows the disease images, and others display the Grad-CAM maps, produced by DAN(Single-Best), DAN(Source-Combine), MFSAN and MDFAN, respectively. It is evident that MDFAN can pinpoint the location of the potato disease more accurately than other methods.

#### 3.3.3 Ablation experiment

Ablation experiments are crucial for understanding the contribution of each component within MDFAN. By isolating the multi-representation extraction module and the subdomain alignment module, we can assess the impact of each part. In the following experiments and analyses, we will explore the significance of these components. Ablation experiments are conducted on transfer tasks corresponding to both 2-source domain and 3-source domain scenarios. **Table 4** presents the experimental results corresponding to the 2-source domain scenario. The compared methods include MFSAN, MDFAN_Inception_, MDFAN_lmmd_, and MDFAN. Among them, MDFAN_Inception_ retains the multi-representation extraction structure, with the subdomain alignment module replaced by MMD; MDFAN_lmmd_, on the other hand, retains the subdomain alignment module but does not have the multi-representation extraction structure.

By examining **Table 4**, it is observed that in 50% of the transfer tasks (Mo,Mi →A; Mo,A →C; Mo,C → A; Mi, A →Mo; Mi,A → C; A,C →Mi), the accuracy of MDFAN_Inception_ and MDFAN_lmmd_ methods is lower than when they are combined, i.e., the accuracy of the MDFAN. This trend is also reflected in the average accuracy metric. It indicates that the combination of multi-representation extraction and subdomain alignment techniques effectively enhances model performance. Moreover, in the remaining 50% of the transfer tasks, the accuracy of MDFAN_lmmd_ is either greater than or equal to the accuracy of MDFAN. Regarding average accuracy, MDFAN_lmmd_ surpasses MDFAN_Inception_ but falls short of MDFAN. This suggests that while subdomain alignment contributes more to the final method’s performance than the multi-representation extraction network, it cannot entirely replace the role of the multi-representation extraction module.

**Table 4:** Classification accuracy (%) of ablation experiments with 2-source domain.

Method	Mo,Mi→A	Mo,Mi→C	Mo,A→Mi	Mo,A→C	Mo,C→Mi	Mo,C→A	Mi,A→Mo	Mi,A→C	Mi,C→Mo	Mi,C→A	A,C→Mi	A,C→Mo	Avg
MFSAN	92.76	84.30	97.14	84.06	94.27	88.32	93.68	80.83	94.36	93.93	95.81	92.78	91.02
MDFAN_Inception_	92.29	83.83	96.03	82.45	93.83	89.72	93.45	79.45	94.58	93.00	95.15	93.68	89.51
MDFAN_lmmd_	92.52	88.68	97.14	83.60	95.37	89.49	94.13	80.14	95.26	95.33	96.04	93.91	91.80
MDFAN	94.39	87.07	96.26	84.30	95.37	90.42	95.03	84.06	94.13	94.63	96.70	93.00	92.11

**Table 5** shows the results of the ablation experiments for the 3-source domain scenarios. In 75% of the transfer tasks (Mo,Mi,A→C; Mo,Mi,C→A; Mo,A,C→Mi), the accuracy of MDFAN_lmmd_ is superior to MDFAN_Inception_ and MDFAN, with MDFAN being the best only in 25% of the tasks. The situation is consistent with average accuracy, where the average accuracy of MDFAN_lmmd_ is higher than that of MDFAN_Inception_ and MDFAN. This indicates that the contribution of the subdomain alignment module is greater than that of the multi-representation extraction module. However, despite this, the average accuracy of the worst-performing method, MDFAN_Inception_, in MDFAN is still better than that of MFSAN.

**Table 5:** Classification accuracy (%) of ablation experiments with 3-source domain.

Method	Mo,Mi,A→C	Mo,Mi,C→A	Mo,A,C→Mi	Mi,A,C→Mo	Avg
MFSAN	85.22	93.93	94.71	93.68	91.89
MDFAN_Inception_	84.99	94.39	95.81	93.23	92.11
MDFAN_lmmd_	87.76	94.63	96.70	93.91	93.25
MDFAN	87.53	94.16	95.81	94.58	93.02

The results of the ablation experiments indicate that both multi-representation extraction and subdomain alignment techniques contribute to improving model accuracy. Specifically, subdomain alignment demonstrated superior effectiveness, suggesting that this technique is more efficient in reducing data distribution differences caused by illumination variations in real field environments. Therefore, compared to other models, MDFAN offers a more effective solution for disease recognition in real agricultural settings.

## 4 Conclusion

This paper proposes a Multi-Source Domain Feature Adaptation Network (MDFAN) based on a two-stage alignment strategy to address the issue of data distribution differences caused by illumination changes in field environments, which negatively impact model performance. In the recognition tasks for five types of potato diseases, MDFAN effectively reduces distribution differences between source and target domains through multi-representation extraction and subdomain alignment techniques, while enhancing prediction consistency. The experimental results demonstrate that MDFAN achieves average accuracies of 92.11% and 93.02% in 2-source and 3-source domain transfer tasks, respectively, significantly outperforming other methods. Furthermore, ablation experiments indicate that the subdomain alignment module contributes more to improving model performance than the multi-representation extraction module, though the combination of both yields the best results.

In our study, the following limitations exist: Although MDFAN demonstrates strong generalization ability under varying lighting conditions, its performance under other differing factors, such as soil conditions, camera types, and crop varieties, as well as its generalization to other crops, still requires further validation. Additionally, the model assumes access to multiple labelled source domain data; however, in practical agricultural environments, collecting and annotating such data may pose significant challenges, particularly under varying field conditions.

Based on the findings and limitations of this study, the following future research directions are proposed: Further exploration of MDFAN’s performance under various environmental factors beyond lighting, such as different soil conditions, crop type variations, and its applicability to other crops, is necessary. Developing a generalized model that can be widely applied across different plant species and disease types will greatly enhance its practical value in agriculture.

Overcoming these limitations will facilitate the deployment of MDFAN in practical applications and further enhance the automation of crop disease recognition.

## Data Availability

The raw data supporting the conclusions of this article will be made available by the authors, without undue reservation.
